# Prevalence and risk factors of work-related musculoskeletal disorders among physical therapists in Ho Chi Minh City, Vietnam

**DOI:** 10.1186/s12889-023-17527-1

**Published:** 2024-01-02

**Authors:** Thao Thi Thach Le, Wattana Jalayondeja, Keerin Mekhora, Petcharatana Bhuuanantanondh, Chutima Jalayondeja

**Affiliations:** 1 Faculty of Physical Therapy & Rehabilitation, Hong Bang International University, 120 Hoa Binh street, Hoa Thanh ward, Tan Phu district, Ho Chi Minh city, Vietnam; 2 Faculty of Physical Therapy, Mahidol University, 999, Phuttamonthon 4 Road, Nakhon Pathom, Salaya District 73170 Thailand

**Keywords:** Health care professional, Job-risk factors, Physical therapy, Occupational health, Work-related musculoskeletal disorders

## Abstract

**Background:**

Understanding risk factors linked to work-related musculoskeletal disorders (WMSDs) is crucial for enhancing health promotion and ensuring workplace safety among healthcare professionals particularly physical therapists (PTs). However, in Vietnam, there has been lack of an investigation. Therefore, this study was to determine whether potential risk factors contributed to the occurrence of WMSDs among PTs in Ho Chi Minh City.

**Method:**

An online self-reported questionnaire for WMSDs comprising the Nordic Musculoskeletal Questionnaire (NMQ), Job-risk and Environmental factors, the Perceived Stress Scale (PSS-4) and the coping strategies, were distributed to PTs. They were enrolled if they had: age ≥ 22 years, graduated from PT program, a full-time job with ≥1 year of experience. Unadjusted and adjusted odds ratios (ORs) with 95% confidence intervals (CIs) were determined using Logistic regression.

**Results:**

Our study found that within the past 12 months, the prevalence of WMSDs was 76.4% (*n* = 204/267): neck 58.4% and lower back 57.3%. PTs aged 22–29 years, < 4 years of education, and < 7 years of working experience were more likely to have WMSDs 2–3 times than those who did not. After adjusting for age, education, and work experience, PTs who engaged in manual techniques/exercises, lifting/transferring patients, and maintaining awkward postures were 5–7 times more likely to have WMSDs in the neck and lower back than those who did not. Environmental and psychological factors, such as number of treatment tables, size of electrotherapy rooms, using PTs modalities, and stress were significantly associated with WMSDs. More than 50% of PTs used modified positions and new treatment/techniques that did not aggravate their symptoms, as coping strategies.

**Conclusions:**

This study indicates potential risk factors associated with WMSDs, affecting the neck and lower back among PTs in Vietnam. These risk factors should be addressed to improve overall PTs health, retain skilled workers, and encourage them to continue working.

## Introduction

Work-related musculoskeletal disorders (WMSDs) are mainly concerned, not only due to their status as a major health problem with consequences for individual workers, but also because of their substantial impact on socioeconomic aspects [1]. WMSDs typically contribute to a significant portion of occupational morbidity, resulting in lost work days, loss of skilled workers and significant increase of economic costs [2]. Health care professionals such as physicians, dentists, technicians, nurses and physical therapists (PTs) are reported to be vulnerable to sustaining occupational health including WMSDs during the course of their work routine [3]. In Vietnam, Luan et al. (2018) conducted a study on nurses and found that the 12-month prevalence of WMSDs was 74.7%, with low back pain (44.4%) and neck pain (44.1%) being frequently reported [4]. Nong et al. (2020) investigated healthcare workers, including physicians, nurses, technicians, pharmacists, and dentists, and found that 62.4% of them experienced WMSDs. The most commonly affected areas were the lower back (48.2%) and neck (40%) [3]. However, there is currently no report on the prevalence and risk factors associated with WMSDs specifically among Vietnamese PTs.

Physical therapy (PT) has gained significant recognition in Vietnam, resulting in a substantial increase in the number of patients seeking treatment from PTs. However, there is a limited workforce of PTs in Vietnam, which poses a challenge in meeting the demand for healthcare services (40,000 population per physical therapist) [5]. This shortage can lead to stress and overwhelm among PTs, negatively impacting their health and potentially giving rise to occupational diseases such as WMSDs within their workplace [6]. Consequently, it would be advantageous to identify all potential risk factors associated with WMSDs among PTs in order to promote health prevention and make policy recommendations within the profession.

Previous studies have identified and categorized common risk factors for WMSDs among PTs worldwide [6–12]. Individual and job-risk factors are mainly influenced on WMSDs occurence in PTs. In the systematic review, Vieira et al. (2015) revealed that WMSDs were highly prevalent among PTs worldwide, reaching up to 90% and at least 50% in those who worked 5 years of PT experience [7]. The most commonly affected area was the low back, attributable to WMSDs [7, 9, 10]. Strong evidence suggests that both individual and work-related factors are significantly associated with WMSDs among PTs [7]. In terms of individual risk factors, female PTs aged ≤30 years with a BMI > 25 kg/m^2^ were more likely to experience WMSDs compared to male who are younger and have lower BMI [11, 12]. Work-related risk factors such as having less than five years of experience, specialization, performing manual therapy techniques, patient transfer, demanding postures, awkward postures, treating a large number of patients, repetitive movements, working while injured, and excessive workload are all significantly associated with WMSDs among PTs (*p* < 0.05) [6–8, 12]. However, there is limited evidence regarding the association between WMSDs and two remaining risk factors: environmental factors and psychological factors among PTs.

WMSDs have a significant impact on PTs, and it is important to explore how they can effectively cope with this situation? Many PTs who have experienced WMSDs reported implementing various reactive/coping strategies such as modifying their techniques, seeking PT treatment, taking medication, consulting doctors, changing their duties, altering clinical habits and work settings, or even leaving their professional positions [6, 8, 13]. However, relying on these coping strategies may not guarantee the sustainability of the PT’s workforce in providing healthcare services [6]. Furthermore, prolonged exposure to this situation can adversely affect their health and quality of life, leading to work inefficiency or early resignation [6, 10].

Therefore, there are two research gaps that need to be addressed. Firstly, lack of the prevalence and risk factors of WMSDs among PTs in Vietnam. Secondly, limited evidence of the association of environmental and psychological factors with WMSDs among PTs. Bridging these gaps and gaining a better understanding of these factors would greatly enhance our approach to preventing and managing WMSDs among physical therapists. Hence, the purpose of this study is to investigate the prevalence of WMSDs and identify potential risk factors contributing to their development among PTs in Ho Chi Minh City (HCMC), Vietnam.

## Methods

This study was an online cross-sectional survey started from February to May 2022. It was approved by the Mahidol University Central Institutional Review Board (MUIRB COA No. 2021/412.2009).

Participants were recruited from government and private hospitals, clinics, and centers in Ho Chi Minh City (HCMC). They were screened using the inclusion criteria as follows: a) Vietnamese individuals aged ≥22 years, b) graduated from a PT program, and c) have a full-time job with at least one year of working experience. They were excluded if they worked in administrative jobs and were not involved with PT clinical practice or unable to work as PTs in the last six months due to pregnancy or illness (including mental, neurological, cardio-pulmonary conditions, or other diseases and injuries).

This study developed an online self-reported questionnaire based on many standardized questionnaires used for WMSD’s survey among PTs [10, 13–18]. It was translated into Vietnamese language using cross-cultural validation and reported elsewhere [19]. An online self-reported questionnaire (the Vietnamese version) had acceptable content validity and test-retest reliability [19]. It consisted of six sections with 25 items as follows. *Section I: individual factor* collects information including age, gender, BMI, level of PT education (vocational, diploma, 3-year/4-year Bachelor’s degree, and postgraduate), sport/exercise duration (min/week), current smoking status (yes/no), monthly income (in USD), working hours, years of working experience, number of patients treated per day, and the specialty of hospital/clinic/center (including orthopedic, neurology, pediatric, cardiopulmonary, or general). *Section II: musculoskeletal pain* asked the participants to rate pain or discomfort in any body part within the last 12 months using the standardized Nordic Questionnaire (NMQ) [14]. If they answered “yes” it referred to having WMSDs and indicated the most pain area by the numeric rating scale (NRS). *Section III: work-related factors* contained 9 items of job-risk factors [15, 16] that can contribute to WMSD among PTs including manual techniques, manual chest PT, exercise, functional activities training, lift/transfer, clerical work, postures/positions, workload, and personal factor. The rating scale represented the significant problems ranging from 1 to 4. The score < 3 was irrelevant, mild to moderate problems and score ≥ 3 was a major problem. *Section IV: environmental factor* comprised of 4 items [17] as follows: number of PTs, number of treatment tables, size of treatment room (including electrotherapy, therapeutic and pediatric rooms) and PT electrical modalities (including ultrasound, TENSE/NMES, LASER, SWD, shockwave and others machines). *Section V: psychological factor* was adopted on the 4-item scale of the Perceived Stress Scale (PSS-4) [18]. Participants were asked to rate their perceived stress over the past month using a 5-point scale ranging from “never” to “almost always.” A score ranged from 0 to 16 and high scores indicate high stress. *Section VI: coping strategies for WMSDs* [10, 13] included eight items for asking participants to rate the effectiveness of coping strategies from “almost always,” “sometimes,” and “almost never.” This section contains a question prompting participants to propose any additional factors they believe might contribute to their work-related musculoskeletal disorders, if relevant.

For data collection, we initially contacted the manager in each setting located in HCMC for permission to invite PTs participating in this study. PTs received a link of survey using Google Forms (Google Inc., Mountain View, CA, USA) via email, Facebook, messenger, Zalo, or text message. It consisted of a self-reported screening questionnaire and an online self-reported questionnaire (Vietnamese version). A total time to administer an online-survey requires 30 minutes and they have two weeks for completing it.

The sample size was estimated using a formula for prevalence study [20]. Based on the previous studies [11, 21, 22], a prevalence of WMSDs was 0.71 among PTs. The significance level (α) was set at 0.05, and the margin of error was set at 5%. After considering for a non-response rate of 20%, the total sample size was 232 participants.

Data were analyzed using the Statistical Package for the Social Sciences (SPSS) version 23.0. Descriptive statistics, including the number and percentage (n, %), mean or median, and standard deviation (SD), were present as demographic data. The association between risk factors and WMSDs was examined through multivariate logistic regression. Both unadjusted and adjusted odds ratios (ORs) were calculated and interpreted with their corresponding 95% confidence intervals (95% CIs). An OR is defined as the strength of association between risk factors and WMSDs occurrence among PTs. The formula is the ratio of the odds of risk factors in the WMSDs and the odds of risk factors in none WMSDs. If OR greater than 1.0 indicated a risk factor and positive associated with WMSDs, while an OR less than 1.0 indicated a protective factor and negative associated with WMSDs. Age, level of PT education, and years of experience were identified as potential confounders based on their statistical significance. A *p*-value was set less than 0.05.

## Results

Three hundred twenty-eight PTs working at 27 government hospitals, 17 private hospitals, 27 clinics and 4 centers in HCMC were screened for eligibility by the questionnaire. Of total, 61 PTs were excluded because they did not graduate from PT program (*n* = 7), less than 1 year of experience in PT practice (*n* = 7), did not a fulltime job (*n* = 38) and unable to work as PT in the last 6 months (*n* = 9) as shown in Fig. 1.267 were enrolled for data analysis. They had 29.5 ± 6.6 years of age and PT experience ranged from 1 to 35 years and provided service for approximately 10 patients per day. Their working hours were 7.9 ± 0.6 hours per day and average 43.1 ± 5.4 hours per week (Table 1).

**Fig. 1:**
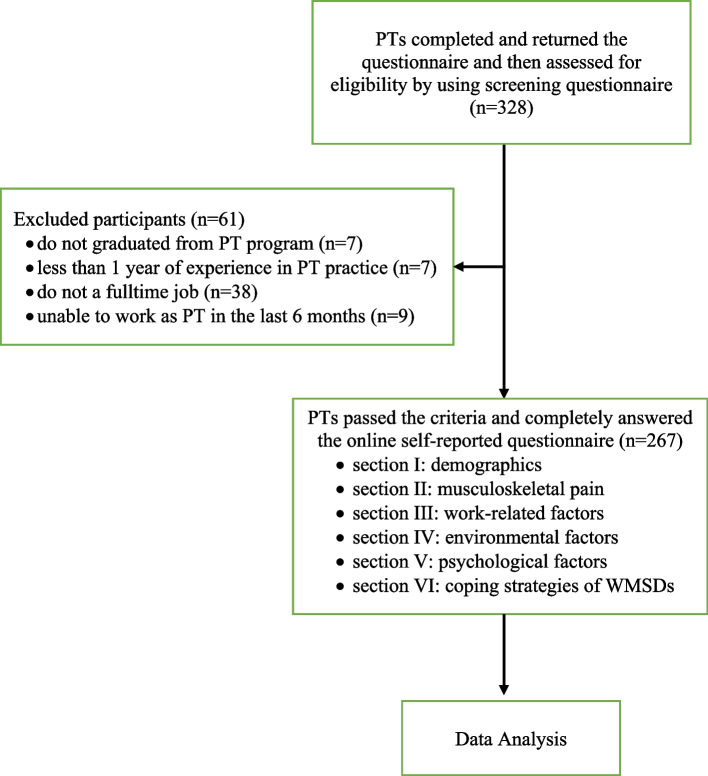
The study flowchart and enrollment

**Table 1:** Demographics and its associated with WMSDs among physical therapists in HCMC, Vietnam (*n* = 267)

Variables	Total (*n* = 267)	WMSDs		OR	95%CI	*p*-value
		Have (*n* = 204) (%)	Not have (*n* = 63) (%)			
Age (years)						
22–29	167	134(80.2)	33(19.8)	3.19	1.33-7.67	0.009^*^
30–39	75	56(74.7)	19(25.3)	2.32	0.90-5.96	0.330
40 and over	25	14(56.0)	11(44.0)	1.00	-	-
Gender						
Male	106	81(76.4)	25(23.6)	1.00	0.56-1.78	0.997
Female	161	123(76.4)	38(23.6)	1.00	-	-
BMI (kg/m^2^)						
Underweight (BMI < 18.5)	15	18(78.3)	5(21.7)	1.26	0.44-3.61	0.674
Normal (18.5 ≤ BMI ≤ 22.9)	147	109(74.1)	38(25.9)	1.00	-	-
Overweight (23 ≤ BMI ≤ 27.5)	82	63(76.8)	19(23.2)	1.16	0.61-2.18	0.653
Obesity (BMI > 27.5)	23	14(93.3)	1(6.7)	4.88	0.62-38.37	0.132
Level of PT education						
4-year Bachelor and postgraduate	199	162(81.4)	37(18.6)	2.71	1.48–4.97	0.001^*^
Vocational/diploma/3-year Bachelor	68	42(61.8)	26(38.2)	1.00	-	-
Sport/exercise (min/week)						
< 150	225	173(76.9)	52(23.1)	1.18	0.56-2.51	0.666
≥ 150	42	31(73.8)	11(26.2)	1.00	-	-
Current smoking status						
Yes	14	8(57.1)	6(42.9)	0.39	0.86-7.74	0.091
No	253	196(77.5)	57(22.5)	1.00	-	-
Monthly income (USD)						
≥ 430 USD	103	81(78.6)	22(21.4)	1.23	0.68-2.21	0.496
Less than 430 USD	164	123(75)	41(25.0)	1.00	-	-
Working hours						
≥ 45 hours/week	175	73(79.3)	19(20.7)	1.29	0.70-2.37	0.412
< 45 hours/week	92	131(74.9)	44(25.1)	1.00	-	-
Year of experience as a physical therapist						
≤ 7	188	151(80.3)	37(19.7)	2.00	1.11-3.62	0.021^*^
> 7	79	53(67.1)	26(32.9)	1.00	-	-
Number of patients treated per day						
> 10	78	65(83.3)	13(16.7)	1.79	0.91-3.54	0.090
≤ 10	189	139(73.5)	50(26.5)	1.00	-	-
The specialty of hospital/clinic/center						
Orthopedics	39	24(61.5)	15(38.5)	0.42	0.19-0.89	0.025^*^
Neurology	34	28(82.4)	6(17.4)	1.21	0.46-3.21	0.702
Pediatrics	28	23(82.1)	5(17.8)	1.19	0.42-3.42	0.743
Cardiopulmonary	2	21(70.0)	9(30.0)	0.61	0.25-1.47	0.265
General	136	108(79.4)	28(20.6)	1.00	-	***-***

*Abbreviations: BMI* Body Mass Index, *CI* Confidence Interval, *OR* Odd Ratio, *PT* Physical therapy, *WMSDs* Work-related Musculoskeletal Disorders

^*^p-value< 0.05

**Fig. 2:**
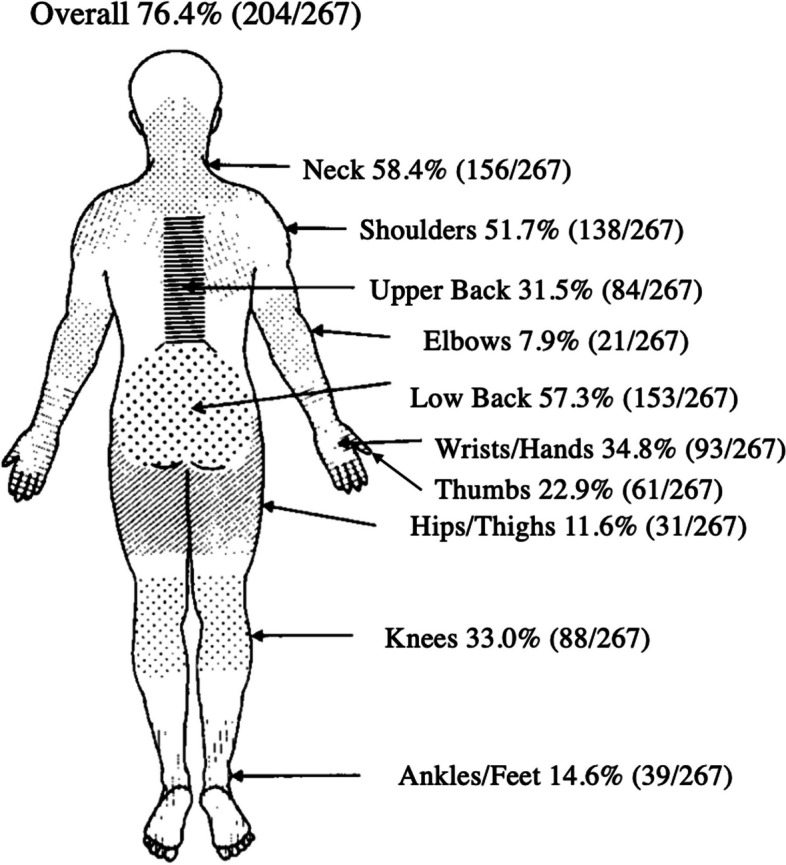
Prevalence of WMSDs in body area

The results showed that the prevalence of WMSDs among Vietnamese PTs in HCMC was 76.4% (*n* = 204/267). The most pain of WMSDs (NRS > 3) were reported in the neck (58.4%), lower back (57.3%), shoulders (51.7%), wrists/hands (34.8%), knees (33.0%), upper back (31.5%), thumbs (22.9%), ankles/feet (14.6%), hips/thighs (11.6%) and elbows (7.9%) as illustrated in Fig. 2.

For individual factors, age, level of education, year of experience as PTs and the specialty of hospital/clinic (*p* < 0.05) were associated with WMSDs among Vietnamese PTs (Table 1). PTs who work at orthopedics settings were less likely to have WMSDs than PTs in general settings 0.42 times (95% CI 0.19 to 0.89, *p*-value = 0.025).

The results showed the association between work-related factors and WMSDs for overall body area among Vietnamese PTs. Nonsignificant association between work-related factors and WMSDs overall area were observed. Except for PTs who used soft tissue technique were more likely to have WMSDs than those who did not (unadjusted OR = 8.71, 95%CI = 1.15–65.63, *p*-value = 0.036). After adjusting for age, education and working experience, the soft tissue work was a risk factor of WMSDs among PTs (adjusted OR = 9.05, 95%CI = 1.19–68.77, *p*-value =0.033).

Tables 2 and 3 demonstrated the work-related factors associated with WMSD at neck and lower back among Vietnamese PTs. The results showed that manual techniques, manual chest PT, exercise, functional activities training, lift/transfer, postures/positions and workload were major contributors to neck and lower back problems among PTs. The unadjusted and adjusted ORs increased the probability of WMSD occurrence at neck related with PTs who performed manual therapy (i.e., joint mobilization, soft tissue work, trigger point release, and segmental breathing), implementing exercise programs (i.e., PROM, performing resistance exercises, manual stretching and PNF techniques), lift or transfer and postures/positions (i.e., maintaining a position for prolonged period of time, bending or twisting in awkward way, squatting or kneeling, and reaching or working away from your body). These findings were also found in the association between work-related risk factors and WMSD at lower back (Table 4). The probability of lower back problems was increased among PTs who performed the same task over and over and continued to work when injured after adjusting for age, education and year of experience.

**Table 2:** Association between work-related factors and neck problem among physical therapists in HCMC, Vietnam

Work-related factor^*a*^		Total (*n* = 267)	The odds of exposures among cases and noncases^*b*^		Unadjusted			Adjusted by age, education and year of experience		
Domain	Items		a/c	b/d	OR	95%CI	*p-value*	OR	95%CI	*p-value*
1. Manual techniques	Joint mobilization	256	11/56	7/182	5.10	1.89–13.79	0.001^*^	7.16	2.36–21.73	0.001^*^
	Soft tissue work	262	15/55	12/180	4.09	1.80–9.26	0.001^*^	4.05	1.78–9.21	0.001^*^
	Trigger point release	252	16/49	10/177	5.78	2.46–13.53	< 0.001^*^	6.13	2.57–14.61	< 0.001^*^
	Massage	244	10/52	14/168	2.30	0.96–5.50	0.059	2.40	0.99–5.80	0.050
2. Manual chest PT	Percussion & vibrations	240	5/57	9/169	1.64	0.53–5.11	0.388	1.72	0.55–5.39	0.351
	Segmental breathing	261	8/63	8/182	2.88	1.04–8.01	0.042^*^	3.08	1.09–8.65	0.032^*^
	Others manual	258	5/65	8/180	1.73	0.54–5.48	0.351	2.08	0.63–6.81	0.223
3. Exercise	PROM	266	11/60	9/186	3.78	1.49–9.58	0.005^*^	3.92	1.53–9.98	0.004^*^
	AAROM	267	50/154	5/190	2.83	0.79–10.10	0.108	2.96	0.80–10.87	0.101
	Resistance exercises	267	13/59	15/180	2.64	1.19–5.87	0.017^*^	2.60	1.16–5.82	0.019^*^
	Passive stretching	267	16/56	14/181	3.69	1.69–8.03	0.001^*^	3.74	1.71–8.19	0.001^*^
	PNF	257	14/55	20/168	2.13	1.01–4.51	0.046^*^	2.13	1.01–4.52	0.049^*^
4. Functional activities training	ADL training	263	6/63	5/189	3.60	1.06–12.20	0.040^*^	3.71	1.08–12.72	0.037^*^
	Gait training	263	5/64	8/186	1.81	0.57–5.75	0.310	2.08	0.64–6.72	0.221
	Stair training	262	7/62	7/186	3.00	1.01–8.89	0.047^*^	3.23	1.07–9.68	0.036^*^
5. Lift/transfer	Lift/transfer	264	17/54	16/177	3.48	1.64–7.35	0.001^*^	3.39	1.60–7.20	0.001^*^
	Adjusting position	267	6/66	13/182	1.27	0.46–3.48	0.639	1.27	0.46–3.50	0.642
6. Work	Clerical work	256	4/65	13/174	0.82	0.25–2.61	0.742	0.81	0.25–2.61	0.725
7. Postures/Positions	Prolonged maintaining position	264	24/48	19/173	4.55	2.30–9.00	< 0.001^*^	4.58	2.31–9.10	< 0.001^*^
	Bending/twisting in awkward way	219	22/47	34/152	2.09	1.11–3.92	0.021^*^	2.01	1.06–3.79	0.032^*^
	Squatting or kneeling	259	22/49	24/164	3.06	1.58–5.94	0.001^*^	3.03	1.53–5.98	0.001^*^
	Reaching away from your body	257	20/51	22/164	2.92	1.47–5.78	0.002^*^	2.86	1.43–5.70	0.003^*^
8. Workload	Performing same task over and over	260	11/60	17/172	1.85	0.82–4.18	0.137	1.85	0.82–4.21	0.137
	Work overtime/irregular shift	255	8/59	22/166	1.02	0.43–2.42	0.959	0.99	0.41–2.36	0.989
	Not enough to take rest of breaks	264	9/62	26/167	0.93	0.41–2.10	0.866	0.88	0.38–2.00	0.764
9. Personal factor	Continuing to work when injured	259	21/50	41/147	1.50	0.81–2.78	0.193	1.43	0.77–2.67	0.255

*Abbreviation:* *AAROM* Active-assisted range of motion exercises, *ADL* Activity of daily living, *PNF* Proprioceptive Neuromuscular Facilitation, *PROM* Passive range of motion exercises

^*a*^The work-related factors which classified into two groups: score ≥ 3 referred to major significant problem and score < 3 referred to irrelevant, mild to moderate significant problems. The participants who reported N/A were not included for data analysis

^*b*^The odds of exposures (score ≥ 3) among neck pain is a/b and the odds of exposures (score ≥ 3) among none neck pain is b/d

^*^p-value < 0.05

**Table 3:** Association between work-related factors and low back pain among physical therapists in HCMC, Vietnam

Work-related factor		Total (*n* = 267)	The odds of exposures among cases and noncases^b^		Unadjusted			Adjusted by age, education and year of experience		
Domain	Items		a/c	b/d	OR	95%CI	*p-value*	OR	95%CI	*p-value*
1. Manual techniques	Joint mobilization	256	6/71	12/167	1.17	0.42–3.25	0.755	1.64	0.55–4.82	0.369
	Soft tissue work	262	14/64	13/171	2.87	1.23–6.45	0.010^*^	2.98	1.31–6.75	0.009^*^
	Trigger point release	252	14/62	12/164	3.08	1.35–7.03	0.007^*^	3.25	1.41–7.51	0.006^*^
	Massage	244	8/67	16/153	1.14	0.46–2.79	0.772	1.16	0.47–2.87	0.742
2. Manual chest PT	Percussion & vibrations	240	6/67	8/159	1.78	0.59–5.32	0.303	1.76	0.58–5.33	0.311
	Segmental breathing	261	7/72	9/173	1.86	0.67–5.21	0.232	1.86	0.66–5.25	0.236
	Others manual	258	6/174	7/174	2.01	0.68–6.46	0.196	2.05	0.64–6.49	0.221
3. Exercise	PROM	266	11/68	9/178	3.19	1.27–8.06	0.014^*^	3.27	1.28–8.32	0.013^*^
	AAROM	267	6/74	4/183	3.70	1.01–13.52	0.047^*^	4.41	1.14–17.04	0.031^*^
	Resistance exercises	267	16/64	12/175	3.64	1.63–8.12	0.002^*^	3.76	1.67–8.48	0.001^*^
	Passive stretching	267	15/65	15/172	2.64	1.22–5.71	0.013^*^	2.77	1.27–6.06	0.010^*^
	PNF	257	16/62	18/161	2.30	1.10–4.81	0.026^*^	2.41	1.14–5.07	0.20
4. Functional activities training	ADL training	263	7/71	4/181	4.46	1.26–15.70	0.020^*^	4.86	1.34–17.60	0.016^*^
	Gait training	263	8/71	5/179	4.03	1.27–12.74	0.018^*^	3.61	1.12–11.62	0.031^*^
	Stair training	262	9/70	5/178	4.57	1.48–14.13	0.008^*^	4.44	1.42–13.91	0.010^*^
5. Lift/transfer	Lift/transfer	264	20/59	13/172	4.48	2.10–9.57	< 0.001^*^	5.16	2.34–11.34	< 0.001^*^
	Adjusting position	267	8/72	11/176	1.77	0.68–4.60	0.236	1.91	0.72–4.98	0.191
6. Work	Clerical work	256	5/73	12/166	0.94	0.32–2.78	0.922	0.96	0.32–2.87	0.948
7. Postures/Positions	Prolonged maintaining position	264	22/58	21/163	2.94	1.50–5.74	0.002^*^	3.04	1.54–5.98	0.001^*^
	Bending/twisting in awkward way	219	22/56	34/143	1.65	0.89–3.06	0.112	1.87	0.98–3.55	0.054
	Squatting or kneeling	259	21/57	25/156	2.29	1.19–4.42	0.013^*^	2.83	1.41–5.68	0.003^*^
	Reaching away from your body	257	19/59	23/156	2.18	1.10–4.30	0.024^*^	2.39	1.19–4.79	0.014^*^
8. Workload	Performing same task over and over	260	14/66	14/166	2.51	1.13–5.56	0.023^*^	2.54	1.14–5.65	0.022^*^
	Work overtime/irregular shift	255	11/67	19/158	1.36	0.61–3.02	0.443	1.42	0.63–3.18	0.390
	Not enough to take rest of breaks	264	14/65	21/164	1.68	0.80–3.50	0.165	1.79	0.85–3.79	0.124
9. Personal factor	Continuing to work when injured	259	26/53	36/144	1.96	1.08–3.55	0.026^*^	2.13	1.15–3.92	0.015^*^

*Abbreviation:* *AAROM* Active-assisted range of motion exercises, *ADL* Activity of daily living, *PNF* Proprioceptive Neuromuscular Facilitation, *PROM* Passive range of motion exercises,

^*a*^The work-related factors which classified into two groups: score ≥ 3 referred to major significant problem and score < 3 referred to irrelevant, mild to moderate significant problems. The participants who reported N/A were not included for data analysis

^*b*^The odds of exposures (score ≥ 3) among lower back pain is a/b and the odds of exposures (score ≥ 3) among none lower back pain is b/d

^*^p-value < 0.05

**Table 4:** Association of environmental and psychological factors with WMSDs among physical therapists in HCMC, Vietnam (*n* = 267)

Risk factors	WMSDs		Unadjusted			Adjusted by age, education and year of experience		
	Have (*n* = 204)	No (*n* = 63)	OR	95%CI	*p*-value	OR	95%CI	*p*-value
**Environment**								
No. PT workforce								
< 12	131	36	1.35	0.76–2.39	0.311	1.44	0.78–2.64	0.243
≥ 12	73	27	1.00	–	–	1.00	–	–
No. of treatment table								
< 12	140	33	1.99	1.12–3.54	0.019^*^	2.32	1.27–4.26	0.006^*^
≥ 12	64	30	1.00	–	–	1.00	–	–
Electrotherapy room								
< 20 m^2^	85	19	1.65	0.90–3.03	0.103	2.13	1.10–4.12	0.024^*^
≥ 20 m^2^	119	44	1.00	–	–	1.00	–	–
Therapeutic room								
< 20 m^2^	53	16	1.03	0.54–1.97	0.926	1.43	0.70–2.90	0.330
≥ 20 m^2^	151	47	1.00	–	–	1.00	–	–
Pediatric room^*a*^								
< 20 m^2^	45	9	1.89	0.80–4.43	0.141	2.18	0.88–5.37	0.089
≥ 20 m^2^	66	25	1.00	–	–	1.00	–	–
Use ultrasound therapy								
Yes	182	45	3.31	1.64–6.69	0.001^*^	3.01	1.46–6.23	0.003^*^
No	22	18	1.00	–	–	1.00	–	–
Use TENSE/NMES								
Yes	170	42	2.50	1.32–4.74	0.005^*^	2.30	1.19–4.46	0.014^*^
No	34	21	1.00	–	–	1.00	–	–
Use LASER								
Yes	79	15	2.02	1.06–3.85	0.032^*^	1.80	0.93–3.49	0.083
No	125	48	1.00	–	–	1.00	–	–
Use SWD								
Yes	136	27	2.67	1.50–4.75	0.001^*^	2.49	1.37–4.52	0.003^*^
No	68	36	1.00	–	–	1.00	–	–
Use shockwave therapy								
Yes	72	14	1.91	0.99–3.69	0.055	1.77	0.90–3.48	0.101
No	132	49	1.00	–	–	1.00	–	–
Use others machines								
Yes	19	8	0.71	0.29–1.70	0.438	0.73	0.30–1.81	0.501
No	185	55	1.00	–	–	1.00	–	–
**Psychological factors by PSS**^***b***^								
> 5.8	91	18	2.01	1.09–3.71	0.025^*^	1.91	1.00–3.60	0.047^*^
≤ 5.8	113	45	1.00	–	–	1.00	–	–

*Abbreviation:* *PSS* perceived stress scale

^*a*^there were 145 physical therapists who reported the area of pediatric room

^*b*^the average of PSS was 5.8 defined as the cut off score

^*^p-value < 0.05

Table 4 demonstrated the results of environmental and psychological factors associated with WMSDs among Vietnamese PTs. Environmental factors including number of treatment tables (< 12), the size of electrotherapy room (< 20 m^2^), PT’s modalities (use of US, TENSE/NMES, LASER and SWD) were significantly associated with WMSDs occurrence among PTs. The result showed that PTs who reported high stress (PSS > 5.8) were 1.91 times more likely to develop WMSDs when compared with those who reported low stress (PSS ≤ 5.8) (95% CI 1.00 to 3.60, *p*-value = 0.047). The unadjusted and adjusted OR of environmental and psychological factors demonstrated the increasing of probability of WMSDs.

**Fig. 3:**
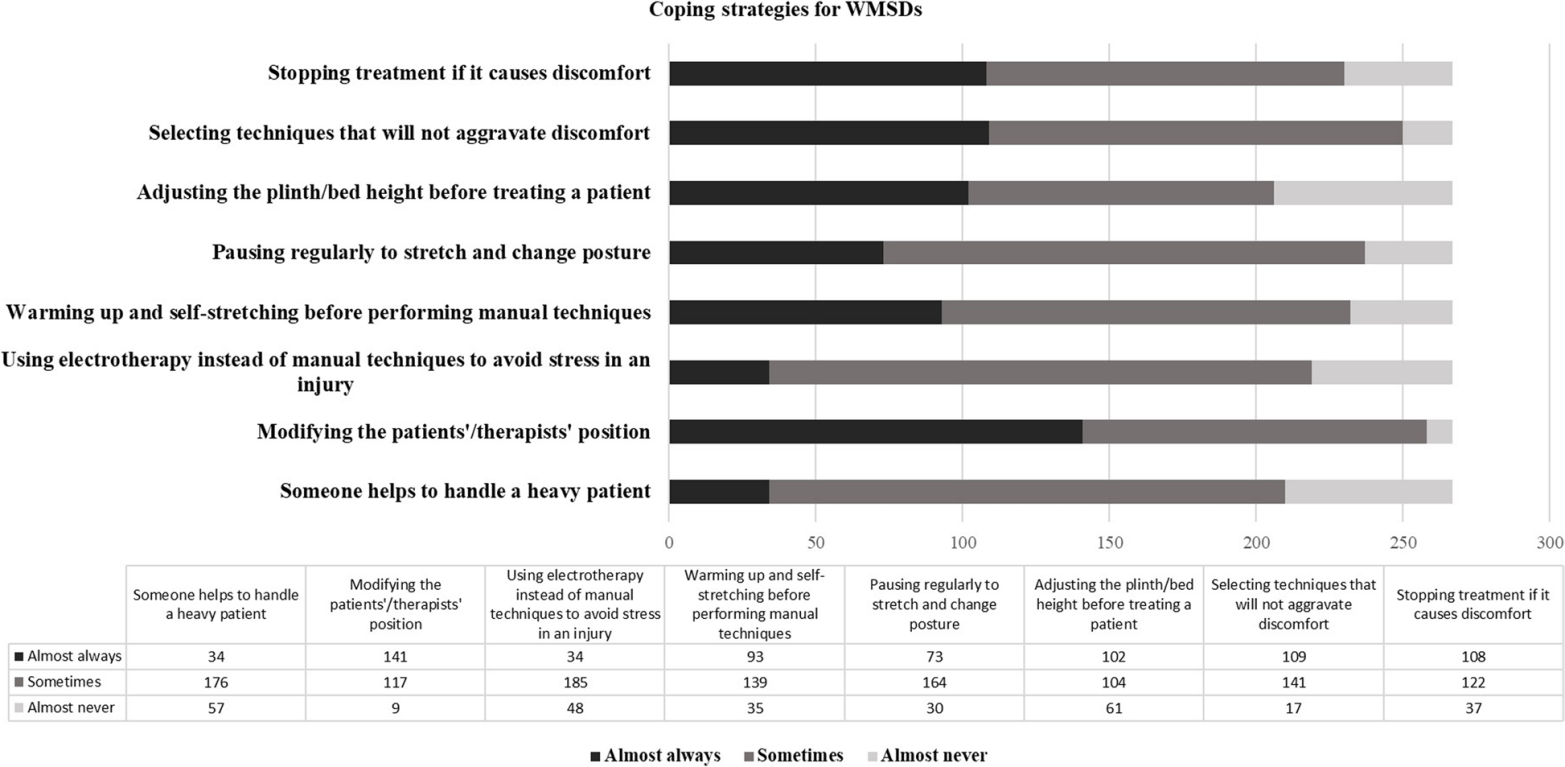
The coping strategies for WMSDs among 267 physical therapists in HCMC Vietnam

The results about the reactive or coping strategies of Vietnamese PTs used for managing WMSDs was illustrated in Fig. 3. To address their WMSDs many of them reported almost always modified their positions or patient positions (52.8%, *n* = 141/267) and halted the treatment which was aggravated their symptoms and selected the new treatment techniques (40.8%, *n* = 109/267). More than 20% of them reported almost never to select the plinth/bed height adjustment before treating patients or call someone to handle a heavy patient as the coping strategies for mitigating their WMSD symptoms.

## Discussion

This study demonstrated a prevalence of WMSDs within a 12-month among PTs in HCMC, Vietnam, reaching up to 76.4%. The most commonly WMSD affected body areas were the neck (58.4%) and low back (57.3%). Corresponding with many countries, they reported the prevalence of WMSDs ranged from 32 to 99.5% [6–13, 23–25]. Our findings corresponded with the prevalence of WMSDs among PTs reported in Southeast Asia [11], which was 71.6%. When compared to the prevalence of WMSDs within a 12-month among other healthcare professions in Vietnam, such as physicians, nurses, technicians, pharmacists, and dentists (ranging from 62.4 to 74.7%) [3, 4], PTs displayed the highest prevalence of WMSDs among health care professions. As a consequence, it is crucial to prioritize the prevention and management of WMSDs as a major concern in order to mitigate the impacts on PTs health and improve occupational health standards in Vietnam.

### The association between individual factors and WMSD among Vietnamese PTs

The results also revealed a significant association of individual factors (i.e., age, level of PT education, year of experience as PT and the specialty of hospital/clinic/center) and WMSDs within 12 months among PTs in HCMC. PTs aged lower than 30 years were more likely to have WMSDs than those who are older which are consistent with many previous findings [12, 13, 15]. Younger PTs may not know how to use self-protection strategies such as modifying treatment techniques, alternating treatment modalities, reducing demanding tasks/activities to alleviate the workload issues and have enough rest breaks between cases [12, 13]. PTs who have less years of experience in PT practice (≤7 years) were more likely to develop WMSDs than those who have> 7 years. Our study found that 97.6% (*n* = 163/167) of PTs aged 22–29 years had less year of experience in PT practice (< 7 years) and 80% of them (*n* = 131/163) reported WMSDs within 12 months. In addition, young PTs with less experience are more prone to make themselves in dangerous positions at work and face a higher risk of WMSDs compared to senior PTs [12, 13, 26].

Interestingly, our findings found increased risk of WMSDs occurence in Vietnamese PTs who graduated higher degree (4 years and more). Because they learned more intensive courses of 4-year Bachelor and postgraduate programs and led to PTs graduated higher academic degrees taking responsibility in multiple tasks in their work more than those who graduated from the 3-year Bachelor and vocational or diploma programs [25, 27]. Additionally, in Vietnam PTs who graduated 4-year of Bachelor’s program learn more in PT techniques such as joint mobilization and PNF techniques that have been exposed to increase the WMSD risk [12, 13, 25]. These reasons lead to an increase in workloads contributing to WMSDs.

The study found that PTs who work at the hospital/clinic/center with orthopedic specialty were significantly associated with WMSDs. Orthopedic PTs were less likely to develop WMSDs when compared with general PTs (OR = 0.42, 95%CI = 0.19–0.89, *p*-value = 0.025). General PTs defined more and multiple workloads/activities than specialist PTs (ie., orthopedic PTs defined as clinical specialists in treatment of the musculoskeletal conditions) and it can be a cause of non-specialized PTs had higher risk to develop WMSDs [13]. Additionaly, our study found that general PTs treated more number of patients than orthopedic PTs (10.7 ± 4.9 patients per day for general setting vs. 8.0 ± 4.3 patients per day for orthopedic setting) which one day working is averaged 8 hours. Therefore, the organizations should redesign workload and schedule as well as recruit more PT workforce to reduce the risk of WMSDs and lost effective workers.

### The association between work-related factors and WMSD at neck and low back among Vietnamese PTs

Our findings found that performing manual therapy, implementing exercise programs, lifting or transferring, postures or positions, workload issues and personal factors were reported by PTs as the major contributing factors for WMSDs at neck and lower back. After minimizing the effect of age, education and year of PT experience, the probability to develop WMSDs at neck and lower back increased twice as much among PTs who were exposed to work-related risk factors. Many previous studies [6, 10, 11] reported that performing manual therapy techniques were the most common work-related risk factors contributing to WMSDs at neck and lower back problems among PTs. Mobilization and soft tissue work and trigger point release techniques are hand-on treatment which can cause of neck, lower back, thumb symptoms [12, 13].

Functional activities training including ADL, gait and stair training were significantly associated with neck and lower back problems among Vietnamese PTs. This might be explained by prolonged standing with lifting or caring patients with frequent twisting and bending when taking care of patients to perform ADL, walking and stair climbing [11]. It is a common cause of neck, upper-limb and lower back problems among PTs.

Lifting or transferring patients and posture/position were the most common cause of neck pain and low back pain in all workers including PTs. Our findings aligned with many previous studies which reported lifting or carrying patients, sustained in the same position or in the awkward twisting position or uncomfortable position, increased risk of neck and lower back problems among PTs [6, 9–11, 13]. Therefore, PTs should be trained and followed preventive strategies at work incorporated with strengthening and flexibility exercise for preventing WMSDs [26].

The previous studies reported that repetitive task and continuing work when having musculoskeletal injuries contributed to more than double times of lower back problem because of prolonged stress of soft tissue [12, 13]. In Vietnam, there are limited number of PT workforce which lead them to response to treat large number of patients per day in various conditions. Although our study found nonsignificant association between number of treating patients per day and WMSDs among Vietnamese PTs (*p* = 0.09), the maximum number of treating patients reached up to 20 patients per day and the average was 9.93 ± 4.79 patients per day. This might contribute to increased clinical workloads and risk of WMSDs among PTs. Consistent with many previous studies [11, 13, 22, 28], Cromie et al. (2000) [13] discovered PTs who treat a large number of patients simultaneously had a statistically significant 2.5 times higher odds of experiencing WMSDs compared to those who did not (95% CI 1.6 to 3.8). Ezzatvar et al. (2020) [22] similarly highlighted that PT who treat a substantial number of patients were 2.14 times more likely to develop WMSDs when compared with those who did not (95% CI 1.53 to 2.99).

### The association between environmental and psychological factors and WMSD among Vietnamese PTs

This study found that environmental factors including number of treatment tables < 12, size of electrotherapy room < 20 m^2^ and using PT electrical modalities were significantly associated with WMSDs among Vietnamese PT in HCMC (*p* < 0.05). Using PT electrical modalities including US, TENSE/NMES, LASER and SWD increased the odds of WMSDs occurrence 2–3 times among PTs. Our findings are contrasted to the previous studies. They reported using electrical modalities are less commonly practice among PTs which might not expose PTs to a high level of risk for WMSDs [11]. Cromie et al. found that injured PTs selected electrotherapy modalities as reactive/coping strategies for self-preservation and enabling themselves to continue working [11, 13]. However, using electrical modalities are most commonly treatment among PTs in Asia particularly in Vietnam. Normally, PTs are assigned to work in an electrotherapy room for one week or month and they might hold the ultrasound transducer for 8 hours of workday or provide various type of PT modalities to consecutive patients. Combined with a high workload of large number of treating patients per day with insufficient number of treatment tables and small treatment room can lead to increase the risk of WMSDs. Therefore, we suggested to modify working scheduling for PTs to avoid performing high workload with a long period.

This study also indicated a notable association between psychological factor including perceived stress and the occurrence of overall WMSDs among PTs in HCMC. This observation aligns with previous studies [18, 28, 29], specifically highlighting that Vietnamese PTs experiencing high stress levels were more prone to the risk of developing WMSDs.

### Reactive or coping strategies used to mitigate risk of WMSDs by Vietnamese PTs

Our study showed the responses about the self-protection to reduce WMSDs symptoms on their body whilst completing work duties. The most coping strategies were modifying the patients’/therapists’ position, selecting techniques that will not aggravate discomfort, stopping treatment if it causes discomfort and adjusting the plinth/bed height before treating a patient. The coping strategies of WMSDs among Vietnamese PTs are the same as those of PTs from other countries [9, 10, 13]. Additionally, to reduce the prevalence of WMSDs among PTs, Campo et al. (2008) [30] proposed that protective measures for lifting or transferring patient should be considered and used suitable equipment such as height-adjustable beds and sliding/lifting equipment. The protevtive measures for performing manual therapy by using assistive devices (e.g., thumb splints, mobilization wedges and instruments assisted soft tissue working) and by considering only applying these techniques on patients who truly needs were also recommended [30]. The role of the Physical Therapy Association is important in formulating and promoting the prevention strategies [30].

Based on Passier and McPhail (2011) [31], Cromie et al. (2001) [32] and the hierarchy of control for improving the work process [33], this study recommended six strategies for prevention WMSDs among PTs. First was an organisational strategy to manage task/workload such as defining PT roles to reduce physical demands and ensuring an appropriate workforce to help. Second was workload arrangement including PTs should take rest of breaks during working or while injuries, regularly perform stretching exercises on targeting muscles affected, and plan an acceptable number of patients treated by PTs per working hour per day. Third, PTs should allow to modify treatment techniques to avoid injuries or aggravate the symptom. Fourth, work setting and provision of equipment suitable for appropriate purpose and sufficient quantities. Fifth focused on improving overall physical health, maintaining a healthy lifestyle, engaging in regular physical activity outside of work, managing stress and having schedule check-ups with professionals to address any health issues especially discomfort or pain. Sixth was education and training PTs can attend workshop or training sessions for proper body mechanics and injury preventions during working.

## Limitations

This study had some limitations that need to be acknowledged. Firstly, being an online self-reported cross-sectional survey, there might have been a potential for recall and information bias among participants. However, to mitigate these issues, we provided clear descriptions and examples in the questionnaire, and participants were encouraged to provide honest responses. Secondly, the data collection occurred during the COVID-19 outbreak in HCMC, Vietnam, which could have influenced respondents’ answers. Some PTs may have experienced changes in their tasks and settings due to the pandemic’s impact. During the three-month lockdown from July to September 2021, PTs were redirected to support the treatment of COVID-19 patients, leading to a high clinical workload and potential challenges in developing WMSDs. Despite these circumstances, we explicitly instructed respondents to base their answers on their usual work-related activities as PTs.

## Conclusion

This study revealed a 12 months prevalence of WMSDs among PTs in HCMC, Vietnam reaching 76.4%. Notably, neck pain and low back pain were the most commonly affected areas. Our investigation comprehensively identified all potential risk factors associated with WMSDs among physical therapists, encompassing individual, work-related, environmental, and psychological factors to promote health prevention and workplace safety recommendations within the profession.

## Data Availability

The datasets generated and/or analyzed during the current study are not publicly available due to organizational confidential but are available from the corresponding author on reasonable request.

## Abbreviations

WMSDs: Work-related musculoskeletal disorders
PTs: Physical Therapists
HCMC: Ho Chi Minh City
BMI: Body Mass Index
PT: Physical Therapy
NMQ: the standardized Nordic Questionnaire
NRS: Numerical pain rating scale
US: Ultrasound
TENS/NMES: Transcutaneous Electrical Nerve Stimulation/Neuromuscular Electrical Stimulation
LASER: Light amplification by stimulated emission of radiation
SWD: Shortwave diathermy
PROM: Passive range of motion exercises
AAROM: Active-assisted range of motion exercises
ADL: Activity of daily living
PNF: Proprioceptive Neuromuscular Facilitation
PSS: The Perceived Stress Scale.
